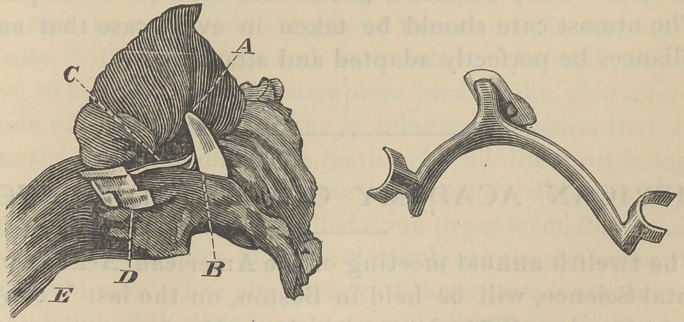# Obituary

**Published:** 1879-10

**Authors:** 


### Editorial.

### OBITUARY.

Died on Friday, August 15th, in this city, Dr. M. B. Wright, aged seventy-two years. Dr. Wright had been longer in medical practice than any physician now living in this city.

There were circumstances attending his last illness and death, of an unusual and peculiar nature, interesting to all, and especially to the members of the dental profession.

Introductory to a presentation of the case, the following remarks of Prof. Comegys before the Academy of Medicine, in this city, and reported in the Lancet and Clinic, of August 23, are given:

"Dr. Comegys remarked that for two years past, Dr. Wright had suffered from occasional attacks of an epileptoid character. They were developed by fatigue, by indigestion, by the supine posture at night, by any severe strain of the mind or body; and on one or two occasions when he was preparing to give a lecture. Nothing had ever been found in the urinary secretion to give them a uræmic character. No coarse lesion had ever been developed in the brain, as he always rallied as bright and clear as ever. It should be stated that he had been subject to attacks of violent palpitation of the heart, and cephalic distress, for over five years before the fits supervened. But nothing indicated that there was any organic lesion of the heart. It always seemed to me as if the convulsions were due to sudden cerebral congestion, dependent on enervation of the vaso-motor nerves, and superinduced by causes referred to.

"Two days before his death, I was requested by Dr. C. O. Wright to see his father with him, who, he stated, had again had convulsive seizures, but at this time there were remaining symptoms of an unusually severe character, such as prolonged unconsciousness, high fever and cough.

"When I reached his bedside, I found that he had already regained consciousness sufficient to recognize me, and he manifested much emotion at my presence. He had a harrassing cough, and I was told that he was spitting some blood; the skin was hot, temp., 104°, pulse 120 and feeble. An examination of the lungs developed dullness over the upper region of right lung, and posteriorly on same side, tubular breathing. There was a crepitant rale posteriorly in the left lung. He complained of pain in right lung alone. From the physical signs, we believed a pneumonitis existed, and he was placed under treatment therefor.

"At the next consultation the following day, we found that the fever had subsided; but the doctor had suffered greatly with his throat all night. It was stated that the only artificial tooth Dr. Wright wore had disappeared, and it was feared that it had gone into the wind-pipe, which would account for the violent cough, streaky sputa, and pain about the larynx; but there was nothing in the respiratory sounds to indicate its lodgment in the larynx or trachea. We believed also that the artificial tooth was too large to have been carried into the lungs, but there existed such pain in the throat, and particularly in an effort at deglutition, that we felt that the missing tooth was in the œsophagus, and Dr. Mussey was called in to explore that tube. He passed his instruments through the whole length of the organ several times, but brought nothing away. The patient felt that the obstacle had been forced downwards, and he said he felt somewhat relieved, but it was still nearly impossible for him to swallow even a teaspoonful of fluid.

"The sufferings of Dr. Wright, before and during the exploration of the œsophagus were simply horrible; his loud cries were heard in the neighborhood. All other conditions of the patient were overwhelmed by his agony in the cervical region.

"From the second visit, he was making such distressing sounds by reason of the pain in the throat, and which were so much transmitted, that it was about impossible to make any satisfactory auscultation.

"It required half a grain of morphia hypodermically, to give him any rest; in that way he obtained about five hour's sleep. Afterwards he grew worse, became unconscious, and gradually sank away."

The following is a portion of the report of the post-mortem made by Prof. J. C. Mackenzie, assisted by Dr. Daniel Young, of the surgical staff of the Hospital, and Dr. Pohlman, interne:

"In the œsophagus was found a plate of gold holding an artificial tooth. The plate was one and five-eigth inches long and one-eigth wide, and was bent so as to form two arms, joining at right angles. The longer one was one inch in length, the shorter five-eighths of an inch. At the angle of junction was fixed the bit of gold holding the tooth, and at the extremities the two catches used by dentists to connect the plate to adjoining teeth. The short arm of the plate lay across the œspohagus, just below the cricoid cartilage; the long arm extended down the tube to a point on a level with the notch of the sternum. The tooth was imbedded in the posterior wall of the œsophagus, through the mucous membrane of which it projected into the subjacent muscular coat. The other organs were not examined."

To add, if possible, to the above clear description the following illustration is given.

In this cut, on the left is shown the œsophagus, as divided opposite the inferior end of the cricoid cartilage with the tooth and plate fixed in the canal just below the point of division.

A represents the gum end of the tooth. This pointed directly forward, and had made quite an impression in the membrane.

B. The cutting edge of the tooth which had made its way through the membrane, and was cutting into the muscular tissue.

C. The short arm of the plate which stood at about a right angle with the course of the canal.

D. The clasp or collar attached to the short arm; this had probably made its way through the wall of the œsophagus, though this was not certainly known, as the excision was made at this point.

E. The downward extension of the œsophagus, into which the long arm of the plate extended.

The figure at the right shows the exact form of the plate with the tooth attached; certainly not a desirable thing to enter and lodge in the œsophagus, or anywhere in the alimentary tract, as might readily have been the case.

This case gives to the dentist a warning that ought to be regarded.

It is not a wonder that this accident occurred, but rather it is remarkable, considering the uncouth and illy adapted fixtures for supporting artificial teeth, that are put into the mouth, that many accidents of this sort have not taken place.

The utmost care should be taken in every case that such appliances be perfectly adapted and attached.

## Figures and Tables

**Figure f1:**